# Treating Aortic Valve Stenosis for Vitality Improvement: The TAVI Study

**DOI:** 10.3390/diseases12080175

**Published:** 2024-08-02

**Authors:** Donato Tartaglione, Dario Prozzo, Renatomaria Bianchi, Giovanni Ciccarelli, Maurizio Cappelli Bigazzi, Francesco Natale, Paolo Golino, Giovanni Cimmino

**Affiliations:** 1 Vanvitelli Cardiology and Intensive Care Unit, Monaldi Hospital, 80131 Naples, Italygiovanni.ciccarelli@ospedalideicolli.it (G.C.);; 2 Cardiology Unit, Cardarelli Hospital, 80131 Naples, Italy; dario.prozzo@gmail.com; 3 Department of Translational Medical Sciences, Section of Cardiology, University of Campania Luigi Vanvitelli, 80131 Naples, Italy; 4 Cardiology Unit, AOU Luigi Vanvitelli, 80138 Naples, Italy

**Keywords:** aortic stenosis, cognitive decline, cerebral flow, quality of life

## Abstract

Background: Degenerative aortic valve stenosis (AS) is the most common valvular heart disease among the elderly. Once cardiac symptoms occur, current guidelines recommend aortic valve replacement. Progressive degeneration/calcification reduces leaflet mobility with gradual cardiac output (CO) impairment. Low CO might induce abnormal brain-aging with cognitive impairment and increased risk of dementia, such as Alzheimer’s disease or vascular dementia. On the contrary, cognitive improvement has been reported in patients in whom CO was restored. Transcatheter aortic valve implantation (TAVI) has proven to be a safe alternative to conventional surgery, with a similar mid-term survival and stroke risk even in low-risk patients. TAVI is associated with an immediate CO improvement, also effecting the cerebrovascular system, leading to an increased cerebral blood flow. The correlation between TAVI and cognitive improvement is still debated. The present study aims at evaluating this relationship in a cohort of AS patients where cognitive assessment before and after TAVI was available. Methods: a total of 47 patients were retrospectively selected. A transcranial Doppler ultrasound (TCD) before and after TAVI, a quality of life (QoL) score, as well as a mini-mental state examination (MMSE) at baseline and up to 36 months, were available. Results: TAVI was associated with immediate increase in mean cerebral flow at TCD. MMSE slowly increase at 36-months follow-up with improved QoL mainly for symptoms, emotions and social interactions. Conclusions: this proof-of-concept study indicates that TAVI might induce cognitive improvement in the long-term as a result of multiple factors, such as cerebral flow restoration and a better QoL.

## 1. Introduction

Life expectancy is improving worldwide, especially in the industrialized countries [1]. As a consequence of population aging, some “age-related” diseases now have time to be detected, because of symptoms or during regular screening. Aortic stenosis (AS) is one of the most common age-related diseases with high prevalence in the population older than 65 years. It is estimated that the burden of AS will further increase in the next 25 years with a prevalence that will double by 2050 [2]. The severe form of AS, if untreated, is associated with high mortality [3]. Because of its structural nature, there are no useful pharmacological treatments. Aortic valve replacement (AVR) is the only effective treatment [4]. For years, surgical replacement has been the only option available [5]. However, in the last decade, tremendous advancements have been made in the treatment of valvular heart diseases, allowing a great improvement in long-term survival [4,5,6]. In this regard, transcatheter aortic valve replacement (TAVR) has become mainstream and proven to be safe in the elderly and in patients at high as well as low surgical risk [6,7]. Thus, the number of TAVR candidates will further increase in the next few years. However, the final therapeutic decision should be made taking into account the clinical characteristics of the patient, the feasibility of the intervention, local experience and outcomes, the individualized surgical risk and how the intervention will improve survival and quality of life [4].

Quality of life (QoL) is becoming an important parameter to be included in most of the clinical studies assessing outcome, with or without treatment [8]. It is a multidimensional concept that includes physical, psychological, cognitive, and social factors that define how a person performs their activities and their self-perceived degree of well-being [8]. It is usually evaluated using a questionnaire that provides a generic measurement of general health, summarizing physical and mental components in items and health domains [9]. Evaluation of QoL in the population with severe AS is still seldom performed and usually limited to the post-surgery (AVR or TAVI) period, or in patients receiving palliative treatment because of the unacceptable risk of surgical intervention. The Toronto Aortic Stenosis Quality of life Questionnaire (TASQ) is a validated tool for AS patients [10,11]. It is well known that the onset of AS symptoms is slow with a long asymptomatic phase [3]. Hence, at the time of evaluation for treatment, most of the patients are elderly, with associated comorbidities [12], often resulting in overlooking the impact of AS on QoL and the need for surgery. However, based on the current guidelines [4], patient-related QoL should be considered during the decision process regarding the best treatment option. In this regard, cognitive status is one of the major QoL components. Cognitive decline may include deficit in memory, attention, orientation, verbal abilities and executive function, thus negatively affecting people’s life in various aspects [13]. Specifically, impaired language may lead to communication difficulties with social consequences; attention deficits may result in daily living problems, such as with eating, bathing and personal care.

Cognitive impairment is a common condition among individuals with AS (ranging from 21 to 39%) [14,15]. Because of the ageing population, the number of AS individuals with dementia is expected to increase worldwide. Decreased cardiac output (CO) because of AS, as well as ageing, atherosclerosis, diabetes, and hypertension, are risk factors involved in cognitive impairment [16]. It has been shown that AVR improves left ventricular stroke volume and carotid artery blood flow [17], as well as cerebral blood flow [18]. It is still to be proven if this increase is also associated with cognitive function improvement. In the present study, we aimed at performing cerebral flow using transcranial Doppler ultrasound, assessing QoL using the Toronto questionnaire and cognitive function by the mini-mental state examination before and after valve replacement.

## 2. Materials and Methods

### 2.1. Patient Selection

This is a retrospective analysis conducted in patients admitted to Vanvitelli Cardiology and Intensive Care Unit—Monaldi Hospital in Naples. Criteria for inclusion were:

(a) Diagnosis of symptomatic or asymptomatic severe AS requiring AVR according to the current guidelines [4], scheduled for TAVI.
(b) Availability of transcranial Doppler (TCD) ultrasound before and after TAVI.
(c) Availability of TASQ score and MMSE before TAVI and in follow-up.

Patients with previous stroke, known carotid disease, severe lung disease, previous AVR or a previous diagnosis of dementia were excluded.

Patients also served as their own controls: baseline measurements in the presence of severe AS were compared with post-TAVI follow-up measurements for all the parameters indicated.

### 2.2. Transcranial Doppler Ultrasonography

Transcranial Doppler (TCD) is an echo-graphic technique that applies Doppler and color Doppler modalities to study the cerebral circulation [19]. This evaluation is routinely used in our center [19,20,21]. It has been performed by placing a low-frequency (≤2 MHz) transducer on the scalp of the patient, in order to visualize the intracranial arterial vessels. Four specific acoustic windows (transtemporal, transorbital, submandibular and suboccipital) may be used for the standard protocol, where bone is thinner [19]. We have used the transtemporal approach as the main window to evaluate cerebral blood flow velocity (CBFV) and its alteration in different cerebrovascular diseases, such as aortic stenosis. In our practice, we mainly use transtemporal and transorbital windows [22]. The transtemporal window allows evaluation of the intracranial carotid artery (ICA) bifurcation with simultaneous flow toward and away from the probe, as the ICA bifurcation terminates in the anterior (flow away from the probe) and middle (flow towards the probe) cerebral arteries (ACA and MCA). The transorbital window can be used to examine the carotid siphon and the ophthalmic artery. TCD was performed in patients lying on the bed during echocardiographic acquisition at admission and before discharge. Peak systolic velocity (PSV) and end diastolic velocity (EDV) were acquired. The mean cerebral blood flow velocity was calculated based on the following formula: (PSV + (EDV × 2)]/3).

### 2.3. Quality of Life Assessment

The TASQ is a 16-item self-administered questionnaire. It requires no more than 5 min to complete. It can be divided in four subscales: physical symptoms (2 items); physical limitations (4 items); emotional impact (7 items); and social limitations (2 items). A single item evaluates health expectations. For each item, participants are asked to rate the current interference of AS on a 7-point scale ranging from “not very much” to “very much”. Items are reverse-coded and summed to compute a total score. Subscale scores may be calculated by summing the reverse-coded items within the subscale. Scores may range from 16 to 112. Higher scores reflect greater perceived QoL. Each of the domains can be scored separately by generating the sum of constituent item responses [11].

### 2.4. Neuropsychological Assessment

For cognitive screening, the Mini-Mental State Examination (MMSE) was used [23]. MMSE is more sensitive for detection of dementia [24,25] and it is considered a reliable tool for a standardized neuropsychological assessment that covers global cognitive functioning and 4 major cognitive domains: memory, executive functioning, attention/psychomotor speed and language [26]. MMSE was performed at admission and during the follow-up (at each visit scheduled and up to 36 months)

### 2.5. Statistical Analysis

All variables were expressed as mean ± SD or percentage of patients. Comparisons between percentages were calculated using the X2 test or Fisher’s test, as appropriate. The differences between means were calculated using the Student’s *t*-test for variables with a Gaussian distribution; otherwise, the non-parametric Mann–Whitney test was employed. All analyses were conducted using the statistical package SPSS 26.0 (IBM, Chicago, IL, USA)

## 3. Results

### 3.1. Patient Population

In our Cardiology Unit, a mean of 100 TAVIs/year are performed (though in 2020 the total number was 33 because of the restrictions applied due to the COVID pandemic). The TASQ score is usually performed and TCD is assessed in the majority of patients as our standard practice [19]. A total of 186 TAVIs performed between 2019 and 2021 were screened. Of these, 47 patients were selected based on the criteria as reported above. The cohort population included 21 males (46%) and 26 females (54%). The middle age was 79.3 ± 4.4. Among the risk factors, 92% of patients were affected by systemic hypertension, 66% by dyslipidemia, and 51% by diabetes. Coronary artery disease was concomitant in about 60% of patients in lines with the data from the available registries. Patients’ characteristics are summarized in Table 1.

### 3.2. Transcranial Doppler Ultrasonography

**Figure 1:**
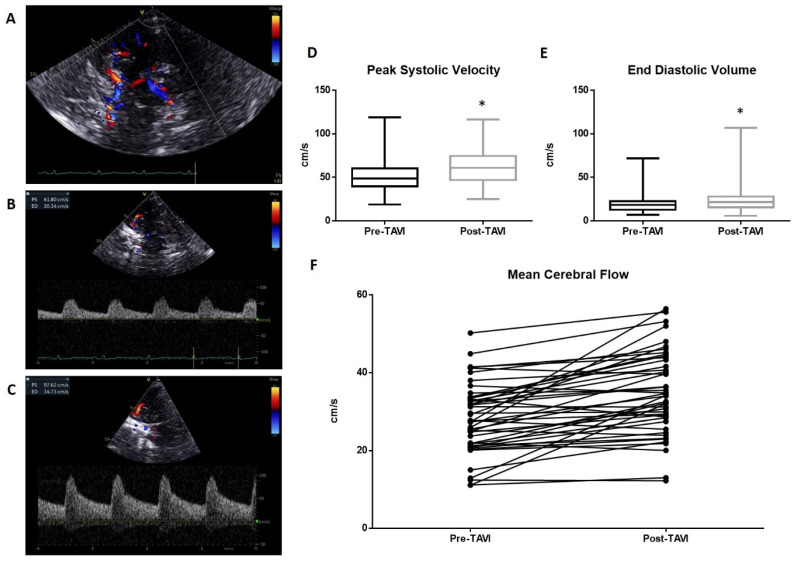
Transcranial Doppler evaluation: (**A**) The transtemporal window with Willis circle visualize at color Doppler. A representative measurement of PSV and EDV is reported in (**B**) (pre-TAVI) and (**C**) (post-TAVI). The average PSV value pre-procedure was 50.21 ± 18.5 cm/s. This value increased significantly (*p* < 0.0001) in the post-procedure measurements, reaching a mean value of 62.9 ± 19 cm/s (**D**). Significant variations occurred in the measurement of End Diastolic Velocity (EDV), which changed from a mean of 20.05 ± 11.1 cm/s to 24.4 ± 16.1 cm/s post-procedure (*p* < 0.005; (**E**)). The resulted mean cerebral flow (MCF) was also significantly improved from 30.11 ± 13.2 cm/s to 37.2 ± 15.9 cm/s (**F**). * = *p* < 0.01; one-way ANOVA with Tukey’s post hoc test.

A representative of TCD evaluation is shown in Figure 1. The transtemporal window (Figure 1A) and Doppler parameters pre- and post-TAVI (Figure 1B and 1C, respectively) are reported. The pre-procedure TCD analysis reveals an average value of peak systolic velocity (PSV) of 50.21 ± 18.5 cm/s. This value increased significantly (*p* < 0.01) in the post-procedure measurements, reaching a mean value of 62.9 ± 19 cm/s (Figure 1D). Significant variations occurred in the measurement of End Diastolic Velocity (EDV), which changed from a mean of 20.05 ± 11.1 cm/s to 24.4 ± 16.1 cm/s post-procedure (*p* < 0.01; Figure 1E). The resulting mean cerebral flow (MCF) was also significantly improved from 30.11 ± 13.2 cm/s to 37.2 ± 15.9 cm/s (Figure 1F).

### 3.3. Quality of Life Assessment

The result of TASQ scores are shown in Table 2.

**Figure 2:**
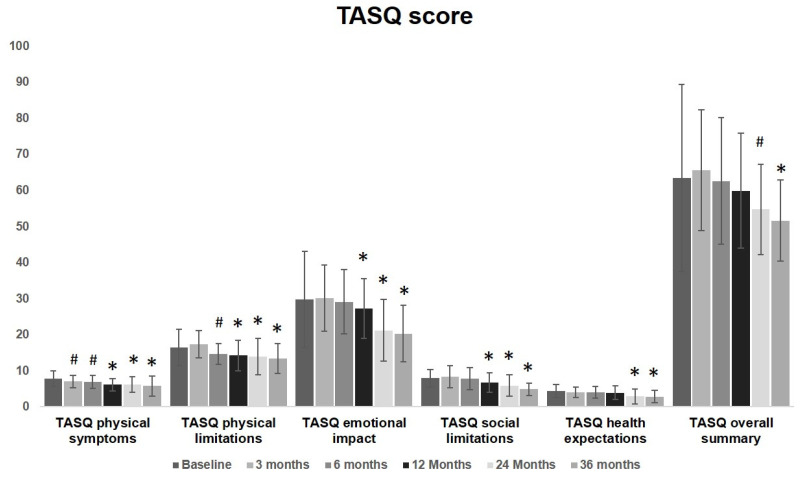
TASQ score graphical representation before and after TAVI: a significant improvement was observed started from 3 months after the procedure for physical symptoms (7 ± 1.7 vs. 7.8 ± 2.1, *p* = 0.04) and from 6 months for physical limitations (14.7 ± 2.9 vs. 16.4 ± 5, *p* = 0.04). These improvements involved physical symptoms, physical limitations and social limitations from 12 months up to 36 months. The overall summary significantly improves at the evaluation performed at 24 months (54.7 ± 12.6 vs. 63.4 ± 25.9, *p* = 0.04), remaining fairly steady at 36 months. TASQ physical limitations and emotional aspects greatly improved over time. Health expectation was almost unmodified up to one year after TAVI (3.9 ± 1.9 vs. 4.4 ± 1.8, *p* = 0.19). Starting from two-years follow-up, a significant improvement was observed (2.9 ± 2.1 vs. 4.4 ± 1.8, *p* < 0.01) up to 36 months. (# = *p* < 0.05, * = *p* < 0.01; one-way ANOVA with Tukey’s post hoc test).

No significant changes were observed in the overall TASQ score during the first 6 months. A significant improvement was observed starting from 3 months after the procedure for physical symptoms and from 6 months for physical limitations. These improvement involved physical symptoms, physical limitations and social limitations from 12 months up to 36 months. The overall summary significantly improves at the evaluation performed at 24 months, remaining fairly steady at 36 months. TASQ physical limitations and emotional aspects greatly improved over time. Health expectation was almost unmodified up to one year after TAVI. Starting from a two-year follow-up, a significant improvement was observed. These findings are graphically reported in Figure 2 to visualize the overall change and the modification of each item over time.

### 3.4. Neuropsychological Assessment

**Figure 3:**
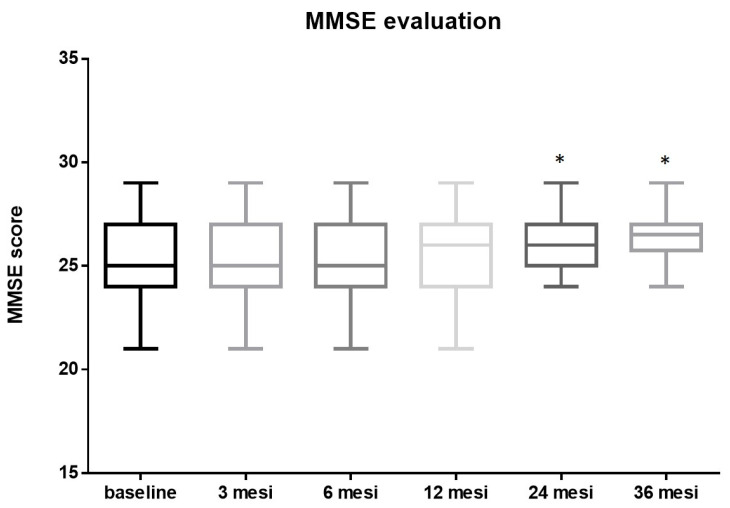
MMSE evaluation: The mean MMSE score before TAVI was 25.15 ± 1.89. No significant changes were observed at 3 (25.22 ± 1.85, *p* = 0.8 vs. baseline) 6 (25.24 ± 1.84, *p* = 0.8 vs. baseline) and 12 months (25.4 ± 1.77, *p* = 0.5 vs. baseline) after the procedure. The MMSE score post TAVI improved significantly by two-years follow-up to a mean of 26.13 ± 1.28, *p* = 0.0047, vs. pre-TAVI, mean Δ 0.98. After this initial rise, the post-TAVI MMSE score remained fairly steady, at around an average of 26.28 ± 1.17, *p* = 0.001, vs. pre-TAVI, mean Δ 1.13 up to three years post-procedure (* = *p* < 0.01; one-way ANOVA with Tukey’s post hoc test).

The initial mean MMSE score before the procedure was 25.15 ± 1.89. No significant changes were observed at 3 (25.22 ± 1.85, *p* = 0.8 vs. baseline) 6 (25.24 ± 1.84, *p* = 0.8 vs. baseline) and 12 months (25.4 ± 1.77, *p* = 0.5 vs. baseline) after the procedure. The MMSE score post-TAVI improved significantly by the two-years follow-up to a mean of 26.13 ± 1.28, *p* = 0.0047, vs. pre-TAVI, mean Δ 0.98. After this initial rise, the post-TAVI MMSE score remained fairly steady, at around an average of 26.28 ± 1.17, *p* = 0.001 vs. pre-TAVI, mean Δ 1.13 up to three years post-procedure (Figure 3).

## 4. Discussion

The present study shows: (1) increased cerebral flow early after TAVI as stated by the TCD evaluation in line with previous reports [18]; (2) improved TASQ score as early as from 3 months after the procedure, mainly for physical symptoms and for physical limitations and social limitations; (3) a significant improvement of MMSE score post-TAVI starting from two years follow-up.

This is the first report on the long-term impact of TAVI in a cohort of patients affected by severe AS evaluating TASQ score and MMSE.

It has been shown that cerebral blood flow is affected by reduced cardiac output (CO) [18,27] and it is improved after its restoration [18]. Accumulated evidence indicates that AS is associated with a decreased cerebral blood flow because of reduced CO [18,28]. age cerebral blood flow declines [27] and brain metabolism resting [29,30] since in elderly brain perfusion becomes increasingly dependent on CO irrespective of the cerebrovascular autoregulation [31]. Hence, restoration of CO may result in an improvement of cerebral blood flow [18]. It is also well known that reduced CO is associated to cognitive impairment in aging adults [32,33,34,35], thus AS may be associated to cognitive decline [36]. Because AS stands for years, progressive cerebral flow reduction occurs, thus influencing cognitive function over the years. It has been already reported that severe cardiac dysfunction, as occurs in severe heart failure, may compromise cerebral blood flow [37,38] which increase after heart transplant [38,39] or if assistant ventricular device is used [40]. Our study, in line with a previous observation [18], indicate that TAVI is associated to a cerebral flow improvement as indicated by the increase PSV and the related MCF.

Symptomatic severe AS is associated with a lower self-reported perception of QoL and our study is in line with previous reports [41]. Symptomatic severe AS significantly impacts lifestyle by limiting the capacity to engage in valued activities, interests, and relationships. In the present study, the TASQ evaluation clearly indicates that symptomatic AS is associated with physical and social limitations, along with a perception of reduced expectation of life. Despite that no significant changes were observed in the overall TASQ score in the first 3 months after the procedure, a substantial improvement was reported for physical symptoms. It is not surprising that patients have still reported physical limitations since, at three months evaluation, groin pain and slow recovery after the procedure were indicated as the main problems. The progressive improvement in symptoms and limitations resulted also in a significant improvement in emotional impact and social activities starting from 12 months after the procedure up to the end of the observation. What remained unchanged were the health expectations up to one-year follow-up and maybe this is not a surprise since, because of age, belief in life end was recurrent. However, at two-years evaluation, a significant improvement was detected, maybe related to better life performance and recovered social activity.

Finally, a slow improvement in MMSE over time was observed. It has already been reported that reduction in cerebral blood flow might impair cognitive function, mainly memory, attention and executive functioning [42]. In line with other previous studies [43,44,45], here we have shown that TAVI is associated with a cognitive improvement of 1 point in the long-term. To our knowledge this is the first analysis evaluating cognitive improvement up to 3 years after TAVI. However, it has to be taken into account that some patients may experience further or new cognitive decline because of silent periprocedural cerebral ischemia [45,46,47]. In the present analysis, early after TAVI we found an increase in cerebral flow. However, if this increase is the main determinant of the observed cognitive improvement, this is still controversial, with some reports supporting this association [18,48], but others not confirming it [49].

Beyond the increased cerebral blood flow reported by this and another study [18], cognitive improvement might be the result of different factors, including recovery from limitations pre-TAVI and engagement in social activities. In this regard, it has been shown that participation in social activities improves cognitive function [50,51]. Compared to the available literature [18], our analysis provides a simultaneous evaluation of this aspect. cerebral blood flow, QoL and cognitive functioning up to 3 years after TAVI. In summary, the results of this proof-of-concept study further support the concept of a relationship between cerebral blood flow and cognitive function in patients with severe AS expanding the previous view since the cognitive improvement might be the resultant of increased cerebral blood flow and the improvement of QoL, mainly the social activities.

In the last decade, percutaneous replacement of the aortic valve has become more reliable with proven efficacy and safety even in low-risk patients [52,53,54]. To date, AVR is recommended in “cardiological” symptomatic AS patients [4]. In the current American and European guidelines, elective AVR in asymptomatic patients is recommended only in selected patients [55]. Left ventricular dysfunction and rapid disease progression and impending onset of symptoms are indicated as additional criteria to evaluate in these patients [55]. Previous observations have also pointed out the role of “non-cardiological” factors in this evaluation [56,57]. Recently, a study has been published showing the therapeutic role of TAVI in patients with anemia because of gastrointestinal angiodysplasia [58]. In this article, it was reported that the number and size of the angio-dysplastic lesions in AS patients were reduced after TAVI, with no further active bleeding detected [58]. The relationship between angiodysplasia and aortic stenosis (AS) is well documented [59]. Moreover, in patients with “asymptomatic” severe AS, anemia significantly affects long-term outcomes [57]. For the first time, it has been reported that a “non-cardiological” symptom significantly improves after TAVI. In line with this paper, our analysis indicated that another “non-cardiological” symptom might improve after TAVI, i.e., cognitive decline.

## 5. Conclusions

The present analysis reveals that TAVI is associated with clinical and cognitive improvement, which is the final result of different aspects (from cerebral flow restoration to QoL). In recent years, growing evidence is expanding regarding “non-cardiological” symptoms and benefits after TAVI. Thus, taking into account the safety of TAVI even in low-risk patients, the role of non-cardiological symptoms should now be considered in the therapeutic flow chart and the next guidelines should consider anemia [57] and angiodysplasia, as well as cognitive decline, as additional criteria for TAVI indication in patients with asymptomatic severe AS.

## 6. Limitations

The current study has some limitations here stated: (1) The retrospective nature; (2) the lack of a control population of AS patients not undergoing TAVI, thus we are missing the natural course of CO and cerebral blood flow in patients with severe AS; (3) it is known that unmeasured factors, such as stress, might influence baseline cerebral blood flow measurement and, despite that this was performed at admission, we cannot exclude this effect; (4) the lack of a brain NMR before and after TAVI to better address the cerebral area involved in cognitive improvement; (5) finally, the lack of specific neurological evaluation, apart from the MMSE evaluation, to better address the degree of cognitive impairment. These results require further validation in a larger-scale trial. In this regard, the CAPITA (CArdiac OutPut, Cerebral Blood Flow and Cognition In Patients With Severe Aortic Valve Stenosis Undergoing Transcatheter Aortic Valve Implantation) study is recruiting AS patients with the main scope of assessing the relationship between CO, cerebral blood flow and cognitive functioning [28].

## Figures and Tables

**Table 1:** Clinical characteristics of the patient cohort.

Study Population (n = 47)	
Demographics	
Age, y	79.3 ± 4.4
Female sex	26 (54%)
Medical history	
Previous myocardial infarction	7
Previous PCI	1
Previous CABG	2
Diabete Mellitus	24
Hypertension	43
Hystory of coronary artery disease	28
Atrial fibrillation	12
GFR < 30 mL/min per 1.73 m^2^	8
NYHA class	I: 3; II: 25; III: 19
Angina pectoris	11
Syncope	3
Medication	
Beta-blockers	24
ACE-inhibitors/ARBs	29
ARNI	15
Diuretics	22
Calcium channel blockers	8
MRA	11
Risk scores	
EuroSCORE II, %	7.8 ± 2.8%
Echocardiographic characteristics pre TAVI	
Aortic maximum gradient, mmHg	69 ± 24
Aortic mean gradient, mmHg	46 ± 8
Aortic valve area, cm^2^	0.6 ± 0.2
SVi, mL/m^2^	36.4 ± 4.2
LVEF, %	41 ± 8
Tricuspid regurgitation	No/trace: 9; mild: 11; moderate/severe: 10
Mitral regurgitation	No/trace: 11; mild: 23; moderate/severe: 6
Aortic regurgitation	No/trace: 12; mild: 15; moderate/severe: 1
Procedural details/valve type	
Transfemoral access	45
Trans-subclavian access	2
Echocardiographic characteristics post TAVI	
Aortic maximum gradient, mmHg	15.6 ± 10.3
Aortic mean gradient, mmHg	9.8 ± 4.2
SVi, mL/m^2^	39.7 ± 6.8
LVEF, %	44 ± 7

Abbreviations: ACE, angiotensin-converting enzyme; ARB, angiotensin II receptor blocker; CABG, coronary artery bypass grafting; CCS, Canadian Cardiovascular Society; EuroSCORE, European System for Cardiac Operative Risk Evaluation; GFR, glomerular filtration rate; LVEF, left ventricular ejection fraction; NT-proBNP, N-terminal prohormone of brain natriuretic peptide; NYHA, New York Heart Association; PCI, percutaneous coronary intervention; SVi, stroke volume index.

**Table 2:** TASQ scores for the study population pre-and after-TAVI.

	Baseline	3 Months	6 Months	12 Months	24 Months	36 Months
TASQ physical symptoms	7.8 ± 2.1	7 ± 1.7	6.9 ± 1.8	6.1 ± 1.7	6.2 ± 2.1	5.8 ± 2.8
TASQ physical limitations	16.4 ± 5	17.3 ± 3.8	14.7 ± 2.9	14.2 ± 4.3	13.9 ± 5.1	13.4 ± 4.2
TASQ emotional impact	29.7 ± 13.3	30.1 ± 9.2	29.1 ± 8.9	27.2 ± 8.3	21.2 ± 8.6	20.2 ± 7.8
TASQ social limitations	7.9 ± 2.5	8.3 ± 3.1	7.8 ± 3	6.8 ± 2.7	5.9 ± 2.9	4.9 ± 1.7
TASQ health expectations	4.4 ± 1.8	4 ± 1.5	4 ± 1.6	3.9 ± 1.9	2.9 ± 2.1	2.8 ± 1.7
TASQ overall summary	63.4 ± 25.9	65.6 ± 16.7	62.6 ± 17.6	59.9 ± 15.9	54.7 ± 12.6	51.6 ± 11.7

## Data Availability

Data and other materials are available from the corresponding author on reasonable request.

## References

[1] Meyer A.C., Drefahl S., Ahlbom A., Lambe M., Modig K. (2020). Trends in life expectancy: Did the gap between the healthy and the ill widen or close?. BMC Med..

[2] d’Arcy J.L., Coffey S., Loudon M.A., Kennedy A., Pearson-Stuttard J., Birks J., Frangou E., Farmer A.J., Mant D., Wilson J. (2016). Large-scale community echocardiographic screening reveals a major burden of undiagnosed valvular heart disease in older people: The OxVALVE Population Cohort Study. Eur. Heart J..

[3] Iung B., Vahanian A. (2012). Degenerative calcific aortic stenosis: A natural history. Heart.

[4] Vahanian A., Beyersdorf F., Praz F., Milojevic M., Baldus S., Bauersachs J., Capodanno D., Conradi L., De Bonis M., De Paulis R. (2022). 2021 ESC/EACTS Guidelines for the management of valvular heart disease. Eur. Heart J..

[5] Rajput F.A., Zeltser R. (2024). Aortic Valve Replacement; Ineligible Companies. Disclosure: Roman Zeltser Declares No Relevant Financial Relationships with Ineligible Companies.

[6] Leon M.B., Mack M.J., Hahn R.T., Thourani V.H., Makkar R., Kodali S.K., Alu M.C., Madhavan M.V., Chau K.H., Russo M. (2021). Outcomes 2 Years After Transcatheter Aortic Valve Replacement in Patients at Low Surgical Risk. J. Am. Coll. Cardiol..

[7] Reyes M., Reardon M.J. (2017). Transcatheter Valve Replacement: Risk Levels and Contemporary Outcomes. Methodist DeBakey Cardiovasc. J..

[8] Haraldstad K., Wahl A., Andenaes R., Andersen J.R., Andersen M.H., Beisland E., Borge C.R., Engebretsen E., Eisemann M., Halvorsrud L. (2019). A systematic review of quality of life research in medicine and health sciences. Qual. Life Res. Int. J. Qual. Life Asp. Treat. Care Rehabil..

[9] Pequeno N.P.F., Cabral N.L.A., Marchioni D.M., Lima S., Lyra C.O. (2020). Quality of life assessment instruments for adults: A systematic review of population-based studies. Health Qual. Life Outcomes.

[10] Styra R., Dimas M., Svitak K., Kapoor M., Osten M., Ouzounian M., Devins G., Deckert A., Horlick E. (2020). Toronto aortic stenosis quality of life questionnaire (TASQ): Validation in TAVI patients. BMC Cardiovasc. Disord..

[11] Arnold S.V., Spertus J.A., Vemulapalli S., Li Z., Matsouaka R.A., Baron S.J., Vora A.N., Mack M.J., Reynolds M.R., Rumsfeld J.S. (2017). Quality-of-Life Outcomes After Transcatheter Aortic Valve Replacement in an Unselected Population. JAMA Cardiol..

[12] Eugene M., Duchnowski P., Prendergast B., Wendler O., Laroche C., Monin J.L., Jobic Y., Popescu B.A., Bax J.J., Vahanian A. (2021). Contemporary Management of Severe Symptomatic Aortic Stenosis. J. Am. Coll. Cardiol..

[13] Stites S.D., Harkins K., Rubright J.D., Karlawish J. (2018). Relationships Between Cognitive Complaints and Quality of Life in Older Adults With Mild Cognitive Impairment, Mild Alzheimer Disease Dementia, and Normal Cognition. Alzheimer Dis. Assoc. Disord..

[14] Lazar R.M., Pavol M.A., Bormann T., Dwyer M.G., Kraemer C., White R., Zivadinov R., Wertheimer J.C., Thöne-Otto A., Ravdin L.D. (2018). Neurocognition and Cerebral Lesion Burden in High-Risk Patients Before Undergoing Transcatheter Aortic Valve Replacement. JACC Cardiovasc. Interv..

[15] Kidher E., Harling L., Sugden C., Ashrafian H., Casula R., Evans P., Nihoyannopoulos P., Athanasiou T. (2014). Aortic stiffness is an indicator of cognitive dysfunction before and after aortic valve replacement for aortic stenosis. Interact. CardioVascular Thorac. Surg..

[16] Han F., Luo C., Lv D., Tian L., Qu C. (2022). Risk Factors Affecting Cognitive Impairment of the Elderly Aged 65 and Over: A Cross-Sectional Study. Front. Aging Neurosci..

[17] van Houte J., Eerdekens R., Dieters E., te Pas M., Wijnbergen I., Tonino P., Bouwman A. (2023). Immediate hemodynamic effects of transcatheter aortic valve replacement on left ventricular stroke volume and carotid artery blood flow. WFUMB Ultrasound Open.

[18] Vlastra W., van Nieuwkerk A.C., Bronzwaer A.S.G.T., Versteeg A., Bron E.E., Niessen W.J., Mutsaerts H.J.M.M., van der Ster B.J.P., Majoie C.B.L.M., Biessels G.J. (2020). Cerebral Blood Flow in Patients with Severe Aortic Valve Stenosis Undergoing Transcatheter Aortic Valve Implantation. J. Am. Geriatr. Soc..

[19] D’Andrea A., Conte M., Cavallaro M., Scarafile R., Riegler L., Cocchia R., Pezzullo E., Carbone A., Natale F., Santoro G. (2016). Transcranial Doppler ultrasonography: From methodology to major clinical applications. World J. Cardiol..

[20] D’Andrea A., Conte M., Riegler L., Scarafile R., Cocchia R., Pezzullo E., Cavallaro M., Di Maio M., Natale F., Santoro G. (2016). Transcranial doppler ultrasound: Incremental diagnostic role in cryptogenic stroke part II. J. Cardiovasc. Echogr..

[21] D’Andrea A., Conte M., Scarafile R., Riegler L., Cocchia R., Pezzullo E., Cavallaro M., Carbone A., Natale F., Russo M. (2016). Transcranial Doppler ultrasound: Physical principles and principal applications in Neurocritical care unit. J. Cardiovasc. Echogr..

[22] Purkayastha S., Sorond F. (2013). Transcranial Doppler Ultrasound: Technique and Application. Semin. Neurol..

[23] Folstein M.F., Folstein S.E., McHugh P.R. (1975). “Mini-mental state”: A practical method for grading the cognitive state of patients for the clinician. J. Psychiatr. Res..

[24] O’Bryant S.E., Humphreys J.D., Smith G.E., Ivnik R.J., Graff-Radford N.R., Petersen R.C., Lucas J.A. (2008). Detecting Dementia with the Mini-Mental State Examination in Highly Educated Individuals. Arch. Neurol..

[25] Mitchell A.J. (2009). A meta-analysis of the accuracy of the mini-mental state examination in the detection of dementia and mild cognitive impairment. J. Psychiatr. Res..

[26] Lansky A.J., Messé S.R., Brickman A.M., Dwyer M., van der Worp H.B., Lazar R.M., Pietras C.G., Abrams K.J., McFadden E., Petersen N.H. (2017). Proposed Standardized Neurological Endpoints for Cardiovascular Clinical Trials. J. Am. Coll. Cardiol..

[27] Jefferson A.L., Liu D., Gupta D.K., Pechman K.R., Watchmaker J.M., Gordon E.A., Rane S., Bell S.P., Mendes L.A., Davis L.T. (2017). Lower cardiac index levels relate to lower cerebral blood flow in older adults. Neurology.

[28] van Nieuwkerk A.C., Hemelrijk K.I., Bron E.E., Leeuwis A.E., Majoie C.B.L.M., Daemen M.J.A.P., Moonen J.E.F., de Sitter A., Bouma B.J., van der Flier W.M. (2023). Cardiac output, cerebral blood flow and cognition in patients with severe aortic valve stenosis undergoing transcatheter aortic valve implantation: Design and rationale of the CAPITA study. Neth. Heart J..

[29] Chen J.J., Rosas H.D., Salat D.H. (2011). Age-associated reductions in cerebral blood flow are independent from regional atrophy. NeuroImage.

[30] Shaw T.G., Mortel K.F., Meyer J.S., Rogers R.L., Hardenberg J., Cutaia M.M. (1984). Cerebral blood flow changes in benign aging and cerebrovascular disease. Neurology.

[31] Bronzwaer A.G.T., Verbree J., Stok W.J., Daemen M., van Buchem M.A., van Osch M.J.P., van Lieshout J.J. (2017). Aging modifies the effect of cardiac output on middle cerebral artery blood flow velocity. Physiol. Rep..

[32] Bown C.W., Do R., Khan O.A., Liu D., Cambronero F.E., Moore E.E., Osborn K.E., Gupta D.K., Pechman K.R., Mendes L.A. (2020). Lower Cardiac Output Relates to Longitudinal Cognitive Decline in Aging Adults. Front. Psychol..

[33] Moore E.E., Jefferson A.L. (2021). Impact of Cardiovascular Hemodynamics on Cognitive Aging. Arterioscler. Thromb. Vasc. Biol..

[34] Ovsenik A., Podbregar M., Fabjan A. (2021). Cerebral blood flow impairment and cognitive decline in heart failure. Brain Behav..

[35] van Nieuwkerk A.C., Delewi R., Wolters F.J., Muller M., Daemen M., Biessels G.J. (2023). Cognitive Impairment in Patients with Cardiac Disease: Implications for Clinical Practice. Stroke.

[36] Solla Suárez P., Díaz R., Herrera J., del Valle R., Moreno C., Almendarez M., López E., Álvarez R., Morís de la Tassa C., Gutiérrez J. (2023). Deterioro cognitivo en el paciente mayor con estenosis aórtica grave sintomática. Toma de decisiones terapéuticas e impacto sobre la mortalidad al año. Rev. Neurol..

[37] Natale F., Golino P., Cimmino G. (2024). Angiotensin-converting enzyme inhibitors, statins and the polypill in cardiovascular diseases prevention: Ignorance is bliss or not?. J. Hypertens..

[38] Gruhn N., Larsen F.S., Boesgaard S., Knudsen G.M., Mortensen S.A., Thomsen G., Aldershvile J. (2001). Cerebral Blood Flow in Patients With Chronic Heart Failure Before and After Heart Transplantation. Stroke.

[39] Massaro A.R., Dutra A.P., Almeida D.R., Diniz R.V.Z., Malheiros S.M.F. (2006). Transcranial Doppler assessment of cerebral blood flow: Effect of cardiac transplantation. Neurology.

[40] Smith K.J., Suarez I.M., Scheer A., Chasland L.C., Thomas H.J., Correia M.A., Dembo L.G., Naylor L.H., Maiorana A.J., Green D.J. (2019). Cerebral Blood Flow during Exercise in Heart Failure: Effect of Ventricular Assist Devices. Med. Sci. Sports Exerc..

[41] Leone A., Gracia Baena J.M., Marsal Mora J.R., Llorca Cardeñosa S., Calaf Vall I., Zielonka M., Godoy P. (2023). Impact of severe aortic stenosis on quality of life. PLoS ONE.

[42] Leeuwis A.E., Smith L.A., Melbourne A., Hughes A.D., Richards M., Prins N.D., Sokolska M., Atkinson D., Tillin T., Jäger H.R. (2018). Cerebral Blood Flow and Cognitive Functioning in a Community-Based, Multi-Ethnic Cohort: The SABRE Study. Front. Aging Neurosci..

[43] Ghanem A., Kocurek J., Sinning J.-M., Wagner M., Becker B.V., Vogel M., Schröder T., Wolfsgruber S., Vasa-Nicotera M., Hammerstingl C. (2013). Cognitive Trajectory After Transcatheter Aortic Valve Implantation. Circ. Cardiovasc. Interv..

[44] Schoenenberger A.W., Zuber C., Moser A., Zwahlen M., Wenaweser P., Windecker S., Carrel T., Stuck A.E., Stortecky S. (2016). Evolution of Cognitive Function After Transcatheter Aortic Valve Implantation. Circ. Cardiovasc. Interv..

[45] Auffret V., Campelo-Parada F., Regueiro A., Del Trigo M., Chiche O., Chamandi C., Allende R., Cordoba-Soriano J.G., Paradis J.-M., De Larochellière R. (2016). Serial Changes in Cognitive Function Following Transcatheter Aortic Valve Replacement. J. Am. Coll. Cardiol..

[46] Pagnesi M., Martino E.A., Chiarito M., Mangieri A., Jabbour R.J., Van Mieghem N.M., Kodali S.K., Godino C., Landoni G., Colombo A. (2016). Silent cerebral injury after transcatheter aortic valve implantation and the preventive role of embolic protection devices: A systematic review and meta-analysis. Int. J. Cardiol..

[47] Ciccarelli G., Renon F., Bianchi R., Tartaglione D., Cappelli Bigazzi M., Loffredo F., Golino P., Cimmino G. (2021). Asymptomatic Stroke in the Setting of Percutaneous Non-Coronary Intervention Procedures. Medicina.

[48] Liimatainen J., Peräkylä J., Järvelä K., Sisto T., Yli-Hankala A., Hartikainen K.M. (2016). Improved cognitive flexibility after aortic valve replacement surgery. Interact. CardioVascular Thorac. Surg..

[49] Lazar R.M., Myers T., Gropen T.I., Leesar M.A., Davies J., Gerstenecker A., Norling A., Pavol M.A., Marshall R.S., Kodali S. (2024). Cerebral blood flow and neurocognition in patients undergoing transcatheter aortic valve replacement for severe aortic stenosis. Eur. Heart J. Open.

[50] Hwang J., Park S., Kim S. (2018). Effects of Participation in Social Activities on Cognitive Function Among Middle-Aged and Older Adults in Korea. Int. J. Environ. Res. Public Health.

[51] Christelis D., Dobrescu L.I. (2020). The causal effect of social activities on cognition: Evidence from 20 European countries. Soc. Sci. Med..

[52] Lim G.B. (2023). Suitability of TAVI in low-risk patients. Nat. Rev. Cardiol..

[53] Shah P.B. (2024). Another Early Win for TAVI in Low-Risk Patients. N. Engl. J. Med..

[54] Forrest J.K., Deeb G.M., Yakubov S.J., Gada H., Mumtaz M.A., Ramlawi B., Bajwa T., Teirstein P.S., DeFrain M., Muppala M. (2023). 3-Year Outcomes After Transcatheter or Surgical Aortic Valve Replacement in Low-Risk Patients With Aortic Stenosis. J. Am. Coll. Cardiol..

[55] Otto C.M. (2022). Alignment and divergence in European and North American aortic stenosis guidelines. EuroInterv. J. EuroPCR Collab. Work. Group Interv. Cardiol. Eur. Soc. Cardiol..

[56] Natale F., Aronne L., Credendino M., Siciliano A., Allocca F., Weizs S.H., Martone F., Marco G.M.d., Calabrò P., Tedesco M.A. (2011). Which is the correct management of patients with asymptomatic severe calcific aortic stenosis after symptomatic spontaneous calcium cerebral embolism?. J. Cardiovasc. Med..

[57] Natale F., Baldini L., Di Marco G.M., Aronne L., Calabro P., Russo M.G., Calabro R. (2010). Management of patients with asymptomatic severe aortic stenosis and severe anemia. Am. J. Cardiol..

[58] Yashige M., Inoue K., Zen K., Kobayashi R., Nakamura S., Fujimoto T., Takamatsu K., Sugino S., Yamano M., Yamano T. (2023). Gastrointestinal Angiodysplasia before and after Treatment of Severe Aortic Stenosis. N. Engl. J. Med..

[59] Sugino S., Inoue K., Zen K., Yashige M., Kobayashi R., Takamatsu K., Ito N., Iwai N., Hirose R., Doi T. (2023). Gastrointestinal Angiodysplasia in Patients with Severe Aortic Stenosis: The Endoscopic Features of Heyde’s Syndrome. Digestion.

